# Combination of 5-Fluorouracil with Photodynamic Therapy: Enhancement of Innate and Adaptive Immune Responses in a Murine Model of Actinic Keratosis^†^

**DOI:** 10.1111/php.13706

**Published:** 2022-09-20

**Authors:** Sanjay Anand, Lauren E. Heusinkveld, Cheng-En Cheng, Lefatshe Lefatshe, Pushpamali De Silva, Tayyaba Hasan, Edward V. Maytin

**Affiliations:** 1 Department of Biomedical Engineering, Lerner Research Institute, Cleveland Clinic, Cleveland, OH, USA; 2 Dermatology and Plastic Surgery Institute, Cleveland Clinic, Cleveland, OH, USA; 3 Cleveland Clinic Lerner College of Medicine, Cleveland Clinic, Cleveland, OH, USA; 4 Department of Inflammation and Immunity, Lerner Research Institute, Cleveland Clinic, Cleveland, OH, USA; 5 Wellman Center for Photomedicine, Massachusetts General Hospital, Boston, MA, USA

## Abstract

We previously showed that a combination of differentiation-inducing agents (5-fluorouracil [5FU], vitamin D3 or methotrexate) and aminolevulinate-based photodynamic therapy (PDT) improves clinical responses by enhancing protoporphyrin IX (PpIX) photosensitizer levels and cell death. Here, we show that in addition to its previously known effects, 5FU enhances PDT-induced tumor-regressing immunity. Murine actinic keratoses were treated with topical 5FU or vehicle for 3 days prior to aminolevulinic acid application, followed by blue light illumination (~417 nm). Lesions were harvested for time-course analyses of innate immune cell recruitment into lesions, *i.e.* neutrophils (Ly6G+) and macrophages (F4/80+), which peaked at 72 h and 1 week post-PDT, respectively, and were greater in 5FU-treated lesions. Enhanced infiltration of activated T cells (CD3+) throughout the time course, and of cytotoxic T cells (CD8+) at 1–2 weeks post-PDT, also occurred in 5FU-treated lesions. 5FU pre-treatment reduced the presence of cells expressing the immune checkpoint marker PD-1 at ~72 h post-PDT, favoring cytotoxic T cell activity. A combination of 5FU and PDT, each individually known to induce long-term tumor-targeting immune responses in addition to their more immediate effects on cancer cells, may synergize to provide better management of squamous precancers.

## INTRODUCTION

The incidence of cancer is consistently increasing worldwide, including in the United States (1). Various cancer treatment modalities including chemotherapy, radiation, hormonal therapy, immunotherapies and photodynamic therapy (PDT) are currently being used, either alone or in combination, for better disease management by reducing cancer progression, metastases and mortality (2). Here, we describe the effects of 5-fluorouracil (5FU), a popular chemotherapeutic drug given either alone or in combination with other agents for the treatment of a range of cancers including colorectal, breast, aerodigestive tract, pancreatic, head and neck and skin (including actinic keratosis and Bowen’s disease) (3,4). The finding that rat hepatoma cells utilize uracil more efficiently than do normal rat intestinal mucosal cells served as the rationale for the synthesis of fluorinated pyrimidines in the 1950s, resulting in successful translation of 5FU into the clinic as an effective chemotherapeutic drug now in use for the past 50+ years (4,5). Although 5FU has been most widely used for colorectal cancer (CRC), its antimetabolite/chemotherapeutic and immunomodulatory tumor-regressing effects have been studied in a variety of cancers in both clinical and preclinical settings for decades (5,6).

### Biochemical mechanisms of action of 5FU

The antimetabolite chemotherapeutic actions of 5FU are achieved by its misincorporation into RNA and DNA and by inhibition of the nucleotide synthetic enzyme *thymidylate synthase* (TS). 5FU, a uracil analog, enters cells by nonfacilitated diffusion and by the same facilitated transport mechanisms used by uracil itself. Subsequently, 5FU is converted to several intermediate metabolites; *fluorodeoxyuridine monophosphate* (FdUMP), *fluorodeoxyuridine diphosphate* (FdUDP)*, fluorodeoxyuridine triphosphate* (FdUTP) and *fluorouridine triphosphate* (FUTP) in cancer cells. These intermediates inhibit DNA and RNA synthesis and the actions of TS. FdUMP is an inhibitor of TS, which is required for *de novo* pyrimidine biosynthesis needed for the proliferation of cancer cells. TS inhibition by 5FU leads to an imbalance of deoxynucleotide pools resulting in misincorporation of FdUTP in genomic DNA, inducing replication stress and double-strand breaks in DNA and triggering apoptotic cell death. In a separate mechanism, FUTP is incorporated into RNA and inhibits normal RNA processing and functions. 5FU misincorporation inhibits preribosomal RNA processing into mature ribosomal RNA and assembly of small nuclear RNA protein complexes, thereby inhibiting the splicing and polyadenylation of messenger RNA [reviewed in detail in (4–7)]. Both FdUTP and FUTP, when misincorporated in DNA and RNA, result in DNA and RNA damage, respectively, causing activation of the tumor suppressor p53 which triggers the elimination of damaged cells by inducing proapoptotic genes and suppressing antiapoptotic genes that primarily belong to the Bcl-2 family of proteins. Several preclinical and clinical studies have established that loss of p53 function, *e.g*. by mutation, reduces the sensitivity to 5FU treatment (4,8).

Acquired resistance following prolonged or repeated treatment with any chemotherapeutic drug can impact the efficacy of treatment; in the case of 5FU, resistance has been reported to involve several different mechanisms. In addition to defects in the pyrimidine synthesis salvage pathway, several *in vitro* and genetic studies have indicated the involvement of: (1) mutation or deletion of enzymes required for 5FU activation; (2) lack of reduced folate substrate; (3) increased activity of catabolic enzymes; and (4) alteration in TS levels by gene amplification, mutation or overexpression [reviewed in (9)].

### Immune response mechanisms of 5FU

Depending on the type and stage of the target cancer and its genomic landscape, a majority of chemotherapeutic drugs induce tumor-eradicating immune responses as their secondary but often equally important curative mechanism (6,10,11). In this section, we discuss the effects of 5FU-based chemotherapy on long-term tumor-regressing immune responses, achieved by reducing immunosuppressive *myeloid-derived suppressor cells* (MDSCs) and stimulating *immunogenic cell death* (ICD) through activation of *damage-associated molecular patterns* (DAMPs). These events ultimately result in the activation of adaptive immunity that involves T cells (6,12,13).

The immune response invoked by 5FU chemotherapeutic regimens can be broadly classified into two categories: (1) reducing or killing immunosuppressive MDSCs; and (2) inducing ICD by activation of DAMPs. The main immune cell types responsible for immunosuppression and escape are: MDSCs, *tumor-associated macrophages* (TAMs), type-II *natural killer T* (NKT) cells and *regulatory T cells* (Tregs) (6,14,15). MDSCs, such as monocytic MDSCs and granulocytic *polymorphonuclear* (PMN) MDSCs, are a mixed population of immature myeloid cells that fail to terminally differentiate into monocytes and neutrophils and that suppress innate and adaptive immune responses. In healthy individuals, MDSCs are present in low numbers and are involved in the maintenance of routine immune surveillance and tissue repair processes. However, during infection, inflammation or cancer, MDSCs rapidly expand and accumulate in the blood, bone marrow, peripheral lymphoid organs and tumors as shown in murine models and in humans (15). Clinical studies show an increase in circulating MDSCs in early and late-stage cancer patients that correlates with stage, metastatic state and therapeutic responsiveness of the disease. By promoting tumor progression, angiogenesis and metastases, MDSCs have been linked with poor prognosis and resistance to routine cancer therapies. For example, melanoma patients who developed progressive disease following checkpoint inhibitor administration had higher baseline levels of MDSCs (16). In preclinical studies, gemcitabine and 5FU have been shown to deplete MDSCs. MDSC depletion induced by 5FU led to increased interferon γ(IFNγ) secretion by tumor-specific CD8+ cells, stimulating a T cell-dependent adaptive immune response (17). Expression of lower levels of TS in MDSCs compared to splenocytes or tumor cells was thought to be partially responsible for the sensitivity of MDSCs to 5FU (17); reviewed in detail in (6,14,15). These findings demonstrate the importance of MDSCs in promoting tumor progression, and the potential for 5FU-based therapies to reduce MDSC levels to improve cancer outcomes.

Cancer cells in response to chemotherapeutic drugs and other cancer therapies undergo different types of cell death including apoptosis, necrosis and as most recently discovered, ICD (12,13,18). ICD leads to the activation of innate and adaptive immune systems, resulting in the eradication of tumors by generating long-term immunological memory. The immune response generated by ICD is dependent on both the antigenicity and adjuvanticity of target cancer cells. Antigenicity of the tumor cells is determined by *tumor-specific antigens* (TSAs), which are cancer-derived epitopes such as neoantigens that can arise through genetic mutations. Adjuvanticity is the cross-presentation of endocytosed TSAs to CD8+ T cells by *dendritic cells* (DCs) *via major histocompatibility class I* (MHC-I). Recruitment of DCs to a dying tumor is usually accompanied by the release of DAMPs that reside in cancer cells as a part of their normal functions, but act as danger signals once released. DAMPs, either secreted or exposed on the extracellular surface of dying cancer cells, are recognized by innate *pattern recognition receptors* (PRRs) such as *Toll-like receptors* (TLRs) expressed on immune cells, thereby promoting the recruitment of *antigen-presenting cells* (APCs). DCs (which are working APCs) endocytose and process the TSAs and then present them to naïve T cells with costimulatory molecules such as CD28 in tumor-draining lymph nodes, leading to the activation of long-term tumor-specific adaptive immunity. Some of the most commonly reported DAMPs include *calreticulin* (CRT)*, high-mobility group box 1* (HMGB1) and *heat shock proteins* (HSPs) 70 and 90, reviewed in detail in (2,6,12,13,19). FOLFOX is an effective regimen for CRC that contains three drugs, 5FU, *oxaliplatin* (OXA) and leucovorin. Whereas 5FU or OXA each induced the release of HMGB1 and HSP70 from cultured CRC cells when given alone, the combination of 5FU plus OXA induced even higher levels of secretion of these particular DAMPs. In the clinic, patients treated with FOLFOX showed higher levels of HMGB1 and HSP70 in their serum, validating the *in vitro* studies (20). Furthermore, media from CRC cell cultures treated with 5FU or with a 5FU plus OXA combination induced maturation of human DCs that was inhibited by a blocking TLR4 antibody (typical and indicative of an innate immune response) (6,20).

Tumors resistant to routine cancer therapies often thrive by upregulating inhibitory genes and pathways that favor tumor growth in an immunosuppressive tumor microenvironment. Therefore, understanding the interaction between a tumor and its host microenvironment is essential for the success of any immunotherapy. Recognition of cancer cells by effector T cells is crucial. However, even in the presence of activated T cells in the immune microenvironment, the antitumor effects of these T cells may be neutralized by upregulation of immune checkpoint molecules such as PD-1, PD-L1 and CTLA-4 that block the cytotoxic effects of T cells, resulting in immunotherapeutic failure (2,6,21,22). *Immune checkpoint inhibitors* (ICI), including antibodies such as pembrolizumab and nivolumab (anti-PD-1), atezolizumab and durvalumab (anti-PD-L1) and ipilimumab (anti-CTLA-4) have been used in the clinical setting for immunotherapy of a variety of cancers (23–25). Combinations of 5FU chemotherapy with ICI have been tried in preclinical studies; success depended upon the types of tumor model, chemotherapy and ICI involved. For example, in a murine CRC tumor model, FOLFOX (relative to monotherapies with 5FU or OXA) resulted in complete tumor eradication when combined with anti-PD-1 treatment (26). The combination of anti-PD-1 with FOLFOX in this study was associated with IFNγ-producing CD8+ T cells and increased expression of PD-L1 on tumor cells, suggesting a link between FOLFOX-mediated antitumor response and the PD-1/PD-L1 pathway (26,27). Today, multiple clinical trials listed on ClinicalTrials.gov are ongoing to explore various combinations of 5FU with immune checkpoint inhibitory agents to enhance treatment outcomes for a variety of cancers (ClinicalTrials.gov). As just one example, a phase II study using FOLFOX with pembrolizumab achieved a favorable response in the *microsatellite stable* marker positive CRC patient population (6,28,29).

### 5FU in combination with photodynamic therapy

PDT for cancer treatment was conceptualized over 100 years ago with the observation that certain dyes (acridine orange and eosin) could cause cell death when exposed to visible light (30). Since the first clinical study of PDT to treat malignant lesions in humans in 1977, PDT has increasingly evolved as a treatment modality for both malignant and nonmalignant conditions, either alone or in combination with other treatment options (2,31–34). PDT, itself a combination treatment, involves the administration of a *photosensitizer* (PS) followed by exposure to light in the presence of oxygen to kill cancer cells. The photoactivation of PS generates *reactive oxygen species* (ROS), including singlet oxygen (^1^O_2_), which results in the destruction of tumor cells *via* different cell death pathways including apoptosis. Depending on the type of PS used, PDT can specifically target lysosomes, mitochondria and/or the *endoplasmic reticulum* (ER) resulting in ROS formation *via* different pathways (35,36). In response to mitochondrial and/or ER photodamage following PDT, autophagy is induced as a cytoprotective mechanism providing a shoulder on dose–response curves (37). Lysosomes therefore could be targeted for better PDT-induced photodamage involving both an apoptotic response and lack of autophagy due to dying lysosomes (37,38). Compared to other treatment options, PDT provides the advantage of dual selectivity for targeting the tumor cells. First, tumor cells metabolize and accumulate PS to higher levels than the normal cells in surrounding tissues. Second, the light source for the activation of PS is focused only on the tumor tissue, enhancing the specificity of the targeted treatment (32,33,39,40). Over many decades, multiple investigators have experimented with different variables and parameters to optimize PDT outcomes. These variables include the type of PS; wavelength and duration of light exposure; oxygen concentration (*vs*. hypoxia) within the tumor; and the physiological state of the tumor (33). Biomodulation of the physiological status of the target tumor by combining PDT with neoadjuvants to alter the state of tumor cell differentiation before PDT is quite promising in both preclinical and clinical studies for the optimization of PDT treatment outcomes (33,41,42).

The first evidence that levels of protoporphyrin IX (PpIX) were linked to the state of differentiation came from a study by Ortel *et al*. (43) using epidermal keratinocytes. Murine epidermal keratinocytes, when induced to differentiate in high levels of calcium in the media, showed elevated levels of PpIX which resulted in enhanced phototoxicity. Later, the concept of combining differentiation-promoting agents prior to PDT to enhance cytotoxic effects was shown in another epithelial cell type, LNCaP prostate cancer cells. Three different types of hormonal agents, *i.e*. androgens, retinoic acid (vitamin A) and vitamin D were used to promote differentiation prior to PDT. All three agents induced higher levels of PpIX accumulation and enhanced phototoxicity when exposed to light. Markers of differentiation and growth arrest were also elevated in these cells, suggesting a link between the state of differentiation of the target cells and higher levels of PpIX and enhanced cytotoxicity (44).

Since the discovery that prodifferentiating agents can be used as neoadjuvants for PDT (a concept called “*combination PDT*” or “cPDT” in our laboratory), we have explored three US Food and Drug Administration (FDA)-approved drugs, *methotrexate* (MTX), *vitamin D* and *5FU* as differentiation-inducing pretreatments prior to PDT (8,45,46). Vit D and 5FU have been successfully translated to the clinic as combination treatment regimens with PDT to better manage cutaneous precancer (actinic keratoses, AK) and *nonmelanoma skin cancer* (NMSC) (47–49). 5FU emerged as a PDT neoadjuvant during the search for a safer alternative to MTX. Although MTX is clearly an effective neoadjuvant for PDT in preclinical models (45,50), it is an oral drug with a documented risk of liver toxicity and is therefore unlikely to be approved by the FDA for this particular indication. 5FU, on the other hand, targets the same pathway as MTX (the salvage pathway for pyrimidine biosynthesis), yet 5FU can be administered as a topical agent. In fact, topical 5FU cream was FDA-approved many decades ago for the treatment of AK. However, although the chemotherapeutic properties of 5FU were already known, its role as a potential neoadjuvant for PDT was completely new. Therefore, we pursued a mechanism-based approach using UVB-induced cutaneous squamous cell carcinoma (SCC) and subcutaneous (A431 and 4T1) murine tumor models. Murine tumors were preconditioned with 5FU either topically or systemically along with vehicle controls, for 3 days. On day 4, tumors were incubated with aminolevulinic acid (ALA) for 4 h followed by exposure to red light (633 nm). In these studies, tumors pretreated with 5FU showed elevated levels of PpIX and enhanced killing after PDT relative to vehicle-pretreated tumors, and two independent mechanisms were identified. First, changes in protein expression levels of two critical enzymes *coproporphyrinogen oxidase* (CPO) and *ferrochelatase* (FC) were identified. Increased expression of CPO and decreased expression of FC, both favoring the higher accumulation of PpIX by increasing PpIX synthesis and reducing PpIX conversion into heme, respectively. At the same time, levels of two other heme-synthetic enzymes, *ALA dehydratase* and *porphobilinogen deaminase* which have been implicated in response to PDT in other cell lines and tumor models, were reduced in 5FU-treated tumors (33). Second, enhanced levels of p53, a gene induced by 5FU and involved in the regulation of growth arrest and apoptosis, were observed in 5FU-treated tumors. Interestingly, PpIX levels were significantly induced by 5FU pretreatment in p53-deficient tumors (A431 and 4T1 tumor models which carry a mutant/defective p53 allele or lack the p53 gene, respectively) (8). This observation may be clinically useful because cancers with a mutant p53 allele often exhibit treatment resistance to 5FU chemotherapy (51). The fact that PpIX levels are enhanced by 5FU even in the setting of p53 deficiency means that PDT is relatively agnostic to p53 status, and therefore a 5FU-PDT combination could be helpful for treating p53-mutant tumors.

AK, which are precancers with the potential to progress to invasive SCC, are treated by dermatologists utilizing a broad variety of therapeutic options including cryotherapy, curettage, topical therapies (5FU, imiquimod, and diclofenac), chemical peels and PDT (52,53). ALA-PDT and 5FU, when applied separately, have given similar clearance response in several clinical trials (54–56). A few studies using a combination of 5FU as a pre- or post-PDT treatment have reported a better lesion clearance rate when 5FU was applied prior to PDT (57–60). Following these reports and to extend our preclinical findings (described above) into the clinical arena, a clinical trial in patients with widespread AK lesions of the face or scalp (a disease for which topical 5FU is FDA-approved) was undertaken (48). In this bilaterally-controlled trial of 17 patients, AK lesions were counted and then 5FU cream (Efudex) was applied daily for 6 days on one side of the face, while the contralateral side served as a PDT-only control. On the day of PDT treatment, methyl ALA was applied and the PpIX level in lesions was measured by noninvasive fluorescence dosimetry or by confocal microscopy in skin biopsies. Relative to control lesions, the 5FU-pretreated lesions showed a two- to three-fold increase in PpIX levels by both measurement techniques. After the dosimetry measurements, PDT was performed using red light, and lesion clearance was measured at follow-up visits every 3 months for 1 year. Lesion clearance at 3 months post-PDT was 75% for 5FU-pretreated lesions *versus* 45% for nonpre-treated lesions, which was a statistically significant difference. Mechanistically, the 5FU-treated AK lesions showed induction of growth arrest and inhibition of proliferation (reduced Ki67), enhanced differentiation (increased E-cadherin), alteration in the expression levels of CPO (increased) and FC (decreased) and induction of p53 by immunofluorescence (48).

Although the therapeutic advantage of combining 5FU with ALA-PDT for treating AKs has been reported by several other groups (57–62), our studies as summarized above provided knowledge about the underlying mechanisms of action when 5FU and ALA-PDT are combined. Thus, we showed that cPDT involving 5FU pretreatment successfully improved the therapeutic response of PDT in preclinical and clinical studies involving SCCs and AKs, and that a central mechanism is induction of differentiation by 5FU which enhances the levels of PpIX accumulation by upregulating heme biosynthetic enzymes, eventually resulting in increased phototoxicity and better lesion clearance (8,48). This, however, is not the whole story. We now report a new mechanism that involves the induction of innate and adaptive immune responses by both 5FU pretreatment and ALA-PDT.

### METHODS

#### UV-induced AK mouse model.

To create an AK model in mice, the dorsal side of SKH-1 hairless female mice at 8 weeks of age was UV-irradiated three times weekly using a set of UV lamps providing 80% UVB and 20% UVA output (63). Due to this chronic UV exposure, lesions of early SCC, which resemble AK lesions both morphologically and histologically, began to appear on the dorsal skin of the mice around week 15 (64). All the experimental procedures were approved by the Institutional Animal Care and Use Committee of the Cleveland Clinic.

#### Photodynamic therapy.

AK lesions were preconditioned with 5FU topical solution (2% w/w from Taro Pharmaceuticals USA Inc., Hawthorne, NY) once daily for 3 days. Petroleum jelly was topically applied as vehicle control. A freshly prepared ALA solution (20% in phosphate-buffered saline with 2% edetic acid disodium salt and 5% dimethylsulphoxide) was topically applied and anesthetized mice were immediately placed under Blu-U^™^ light source (Sun Pharmaceuticals, Mumbai, India; 417 nm, 60 min, 36 J cm^−2^) in a regimen designed to mimic the clinical protocol currently used for “painless PDT” (65), but adapted here for hairless mice (64). Mice were sacrificed and lesions were harvested at various times post-PDT as shown in the figures.

#### Immunofluorescence staining of AK samples.

Formalin-fixed paraffin-embedded sections were evaluated by immunofluorescent staining for immune cell markers. Primary rabbit antibodies to neutrophils (Ly6G), macrophages (F4/80), T cells (CD3 and CD8) and PD1 were from Cell Signaling Technologies (Danvers, MA). The secondary antibody, Cy3-conjugated donkey antirabbit IgG (excitation peak at 555 nm and emission peak at 569 nm) was from Jackson ImmunoResearch Laboratories (West Grove, PA). Relative expression of marker proteins in immunofluorescently stained sections was analyzed using fluorescence microscopy. Multiple images were captured digitally, and the number of positively stained cells was counted and expressed as cells per high power field. Data presented in the graphs in the figures represent the analysis of three immunofluorescent images from each of five lesions per condition (harvested from at least three different mice), for each treatment group examined in the time course.

#### Statistical analysis.

Cell counts per 20X field from immunofluorescence images were analyzed using GraphPad Prism (GraphPad Software, San Diego, CA). Data sets were first tested for Gaussian (normal) distribution, and then analyzed using the appropriate approach, *i.e*. by analysis of variance and unpaired two-sided t-tests if normally distributed, or by a nonparametric test (Mann–Whitney) if not normally distributed. *P* values <0.05 were considered statistically significant.

## RESULTS AND DISCUSSION

### Additive effect of 5FU and PDT on induction of innate immunity by infiltration of neutrophils and macrophages in murine AK lesions

**Figure 1.**
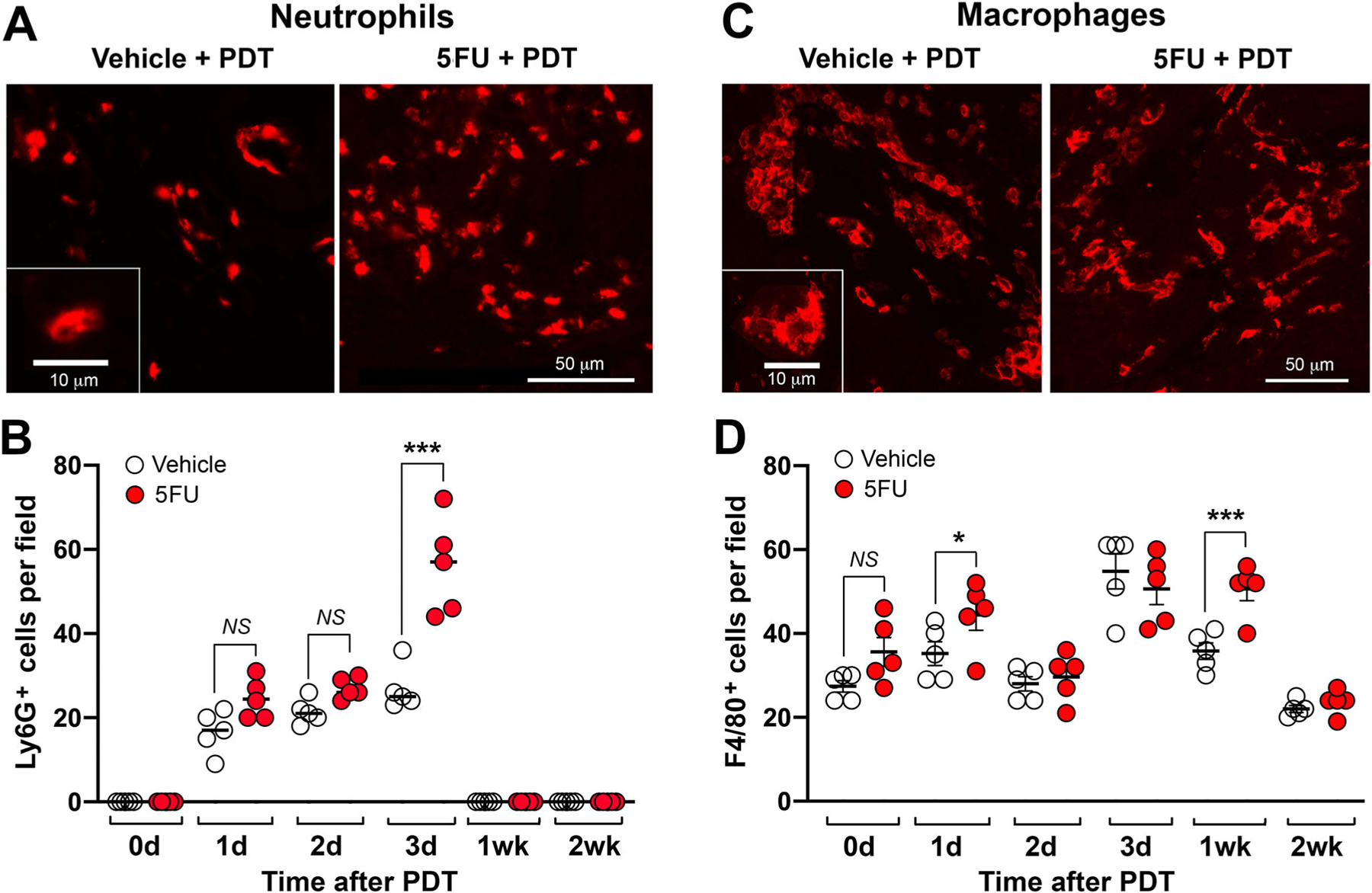
Quantification of innate immune cells (neutrophils and macrophages) in murine AK lesions at various times after PDT ± 5FU. (A) Immunofluorescence images showing infiltration of neutrophils stained for Ly6G, at 72 h after PDT. *Inset*, an enlarged image showing morphology of a neutrophil. (B) Neutrophil counts from five independent AK lesions per condition, reported per high-power field, at each time during the post-PDT treatment time course (*d*, days; *wk*, weeks). (C) Immunofluorescence images of macrophages in AK lesions, stained for F4/80, at 72 h after PDT; an enlarged image shows the morphology of a typical macrophage. (D) Enumerated macrophages are reported per high-power field at each time during the post-PDT time course. In (B) and (D), the significance of differences between 5FU-pretreated *versus* vehicle-pretreated lesions is indicated above the brackets: NS, not significant; *P < 0.05; ***P < 0.005 (unpaired two-sided Student’s t-test).

The main therapeutic effect of conventional PDT, in which exposure to light is preceded by several hours of ALA preincubation, is assumed to result from tumor cell death mediated by the production of ROS during light exposure. However, in our recent studies of simulated daylight PDT, also called “painless PDT” because ALA incubation and light exposure occur simultaneously, providing a pain-free experience for the patient (65), we showed that immediate cytotoxicity was not the primary mechanism of lesion clearance, despite a similar lesion clearance rate observed with the painless and conventional regimens (64). Instead, activation of innate and adaptive immune events within PDT-treated lesions was demonstrated (64). Here, since 5FU and PDT are both immunogenic, we compared the induction of innate immunity in murine AK lesions after PDT ± 5FU pretreatment in a time course analysis. Mice with AK lesions were treated topically either with vehicle or 5FU, followed by the application of ALA and immediate exposure to blue light. Some lesions were harvested prior to PDT to analyze the effect of 5FU treatment alone. Lesions treated with PDT were harvested at 24, 48 and 72 h, and 1 and 2 weeks post-PDT for analysis of the immune response by immunofluorescence staining of tissue sections using antibodies against neutrophils and macrophages. Infiltration/recruitment of neutrophils (stained by Ly6G antibody; Fig. 1A) was observed at 24, 48 and 72 h post-PDT-treated samples (Fig. 1A,B), compared to a complete absence of neutrophils before PDT or at 1 and 2 weeks posttreatment. A trend toward higher neutrophils in 5FU-pretreated lesions was evident at 1 and 2 days after PDT, but this difference became prominent and significant (two-fold) at 72 h post-PDT (Fig. 1B). In conventional PDT using photofrin as the PS, Krosl *et al*. (66) showed that neutrophils were the most predominant leukocytes that accumulated in murine SCC tumors, starting within minutes after PDT and reaching a maximum at 24 h post-PDT. In painless PDT, which involves ICD instead of apoptotic cell death as the primary mechanism, a delayed peak (72 h post-PDT) is observed in murine AK lesions as shown in Fig. 1B (2,64). Relative to PDT alone, higher numbers of neutrophils present in 5FU-pretreated/PDT-treated lesions should favor maturation and activation of DCs, which then trigger adaptive immunity by stimulating CD8+ T cells.

Macrophages (stained by F4/80 antibody; Fig. 1C) were present in vehicle-treated lesions and were slightly increased (1.3-fold) in 5FU-treated lesions, even before PDT (Fig. 1D). Macrophage numbers increased modestly after PDT during the time course, but more interestingly, macrophages increased significantly more in 5FU-pretreated lesions at the 24-h (1.3-fold) and 1 week (1.4-fold) time points when compared to PDT alone (Fig. 1D). These findings have several possible implications. TAMs, an important component of the *tumor microenvironment* (TME), differentiate from monocytes and regulate immune effector functions after PDT. In an unperturbed immune-suppressive TME, most TAMs belong to the tumor-promoting “M2” subtype, which is anti-inflammatory and able to promote immunosuppression, tumor growth, angiogenesis and metastases. Following PDT, the majority of M2 macrophages are replaced by fresh M1 macrophages which are derived from monocytes present in the tumor and surrounding vasculature (2,67). These M1 macrophages serve as the main source of proinflammatory and immunostimulatory signals, primarily *via* secretion of proinflammatory cytokines, *e.g*. IL-1, IL-6, IL-12 and TNFα, which promote tumor eradication (2,64,68,69). It is important in future work to investigate which type of macrophage (M1 *vs*. M2) is associated with the increases seen in the presence of 5FU during the time course, and whether 5FU pretreatment ± PDT results in a switch or polarization in macrophages phenotype (70).

### Enhanced infiltration of different T cell populations in murine AK lesions by 5FU and PDT

**Figure 2.**
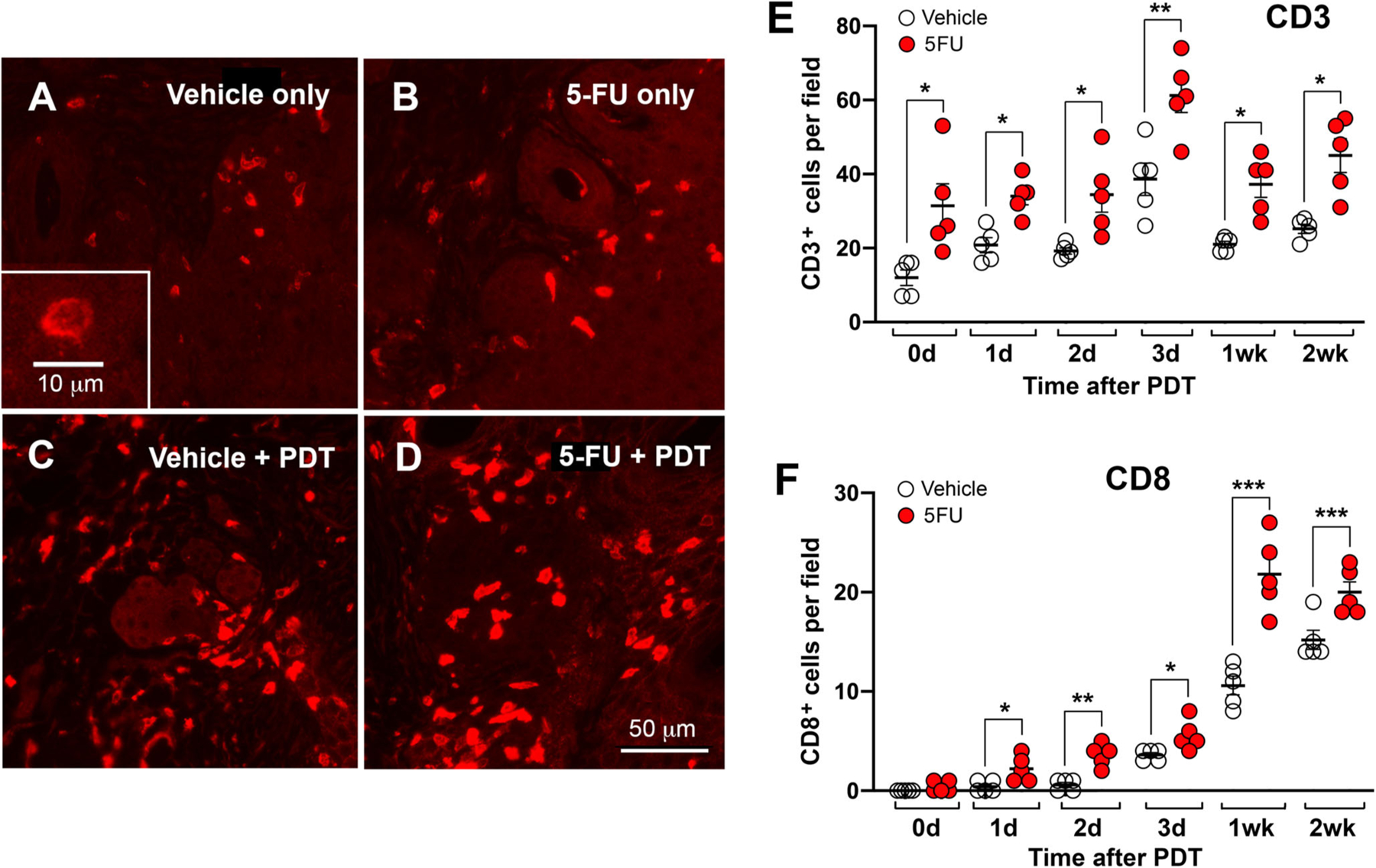
Quantification of T cells involved in the adaptive immune response in AK lesions at various times after PDT ± 5FU. Immunofluorescence images showing recruitment/infiltration of T cells in murine AK lesions in response to PDT, with or without 5FU pretreatment. In (A–D), immunofluorescence images of CD3+ active T cells (stained for CD3?) in AK lesions, either just before PDT (A, B), or 72 h after PDT (C, D). Immunostaining for CD8+ cytotoxic T cells (stained for CD8α in AK lesions) looked qualitatively similar. In the graphs, CD3+ cells (E) or CD8+ cells (F) were counted in AK lesions harvested during a time course experiment and reported as cells per high-power field. Note the statistically significant increases in T cell counts caused by 5FU pretreatment, both before PDT and more so after PDT, as indicated by asterisks above the brackets (unpaired two-sided Student’s t-test): *P < 0.05; **P < 0.01; ***P < 0.005.

Activation of innate immunity triggers induction of adaptive immune responses that require interaction between APCs and T cells; this interaction results in the proliferation of T cell clones which recognize specific epitopes on the target tumor (2,12,32,71). To investigate the activation of adaptive antitumor immunity in murine AK lesions after PDT, the infiltration of different T cell populations was analyzed by immunofluorescence using antibodies against CD3 (pan-activated T cell marker) to evaluate the overall number of T lymphocytes. Prior to PDT, a few CD3+ T cells were present in vehicle-treated lesions, and 5FU treatment led to an increase in overall T-cell infiltration (Fig. 2A,B) of ~2.6-fold (Fig. 2E). Following PDT, the number of infiltrating CD3+ T cells was enhanced (Fig. 2C,D) and similar magnitude of increase was observed at all times post-PDT, averaging 1.6- to 1.8-fold (Fig. 2E). Although induction of adaptive immunity by T cell infiltration post-PDT has been shown by us and others (2,64,72), the main goal here was to analyze the effect of 5FU-PDT on T cell infiltration.

Interaction between APCs and naïve T cells results in the development and differentiation of different subclasses of T cell subsets. Antitumor adaptive immune responses following PDT mainly involve CD4+ *T helper* (Th) cells, CD8+ *cytotoxic T lymphocytes* (CTLs) and Tregs, as reviewed in reference (2). As seen previously with adaptive immune response in painless PDT-treated murine AK lesions (64), CD8+ T cells were dramatically affected by PDT, with the majority appearing at 1 and 2 weeks post-PDT (Fig. 2F). Interestingly, 5FU pretreatment caused sizeable increases (more than two-fold) in the number of CD8+ T cells at all timepoints after PDT, relative to PDT alone, and these were all statistically significant differences (Fig. 2F). Overall, the findings of an enhanced presence of CD3+ and cytotoxic CD8+ T cells in PDT-treated AK lesions after a 5FU-PDT combination, relative to PDT alone, suggests an important tumoricidal benefit of the combination approach. CD8+ T cells appear to play a functional role in tumor eradication induced by PDT. Korbelik *et al*. (73) demonstrated that depletion of CD8+ T cells in an EMT6 mammary carcinoma murine model resulted in a 50% decrease in tumor eradication relative to controls. In another study, adoptive transfer of CD8+ T cells from PDT-treated mice protected naïve recipient mice from developing tumors of the same origin (74). Since 5FU and PDT are both known to induce ICD by activation of DAMPs, we postulate that an enhanced adaptive immune response (as suggested by higher numbers of CD3+ and CD8+ T cells) might be the downstream result of ICD that was triggered preferentially by 5FU and PDT in these lesions. Additionally, suppression of MDSCs (a prominent tumor-regressing mechanism of 5FU) could also have a positive effect on adaptive immunity mediated by T cells (6). Further work is necessary to assess peripheral and tumor-infiltrating levels of PMN MDSC’s and monocytic MDSC’s pre and post-PDT with 5FU. These results would help determine the importance of a PDT-induced inflammatory environment on the induction and programming of MDSC subsets.

### 5FU suppresses PDT-induced expression of the immune checkpoint marker PD-1 in murine AK lesions

**Figure 3.**
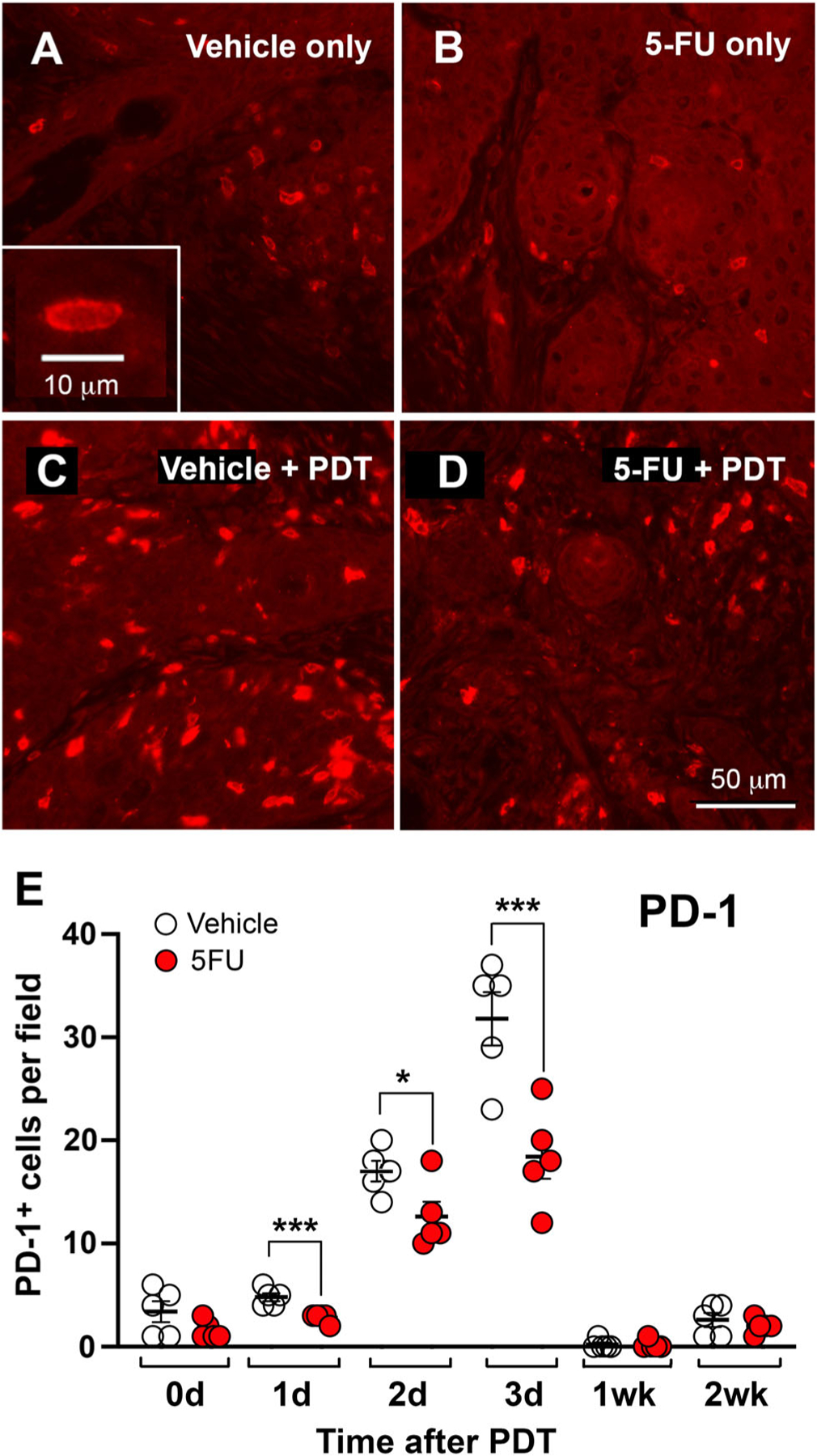
Suppression by 5FU of PD-1 expression in immune cell populations in murine AK lesions after PDT. (A–D) Histological sections of AK lesions, stained with an anti-PD-1 antibody either just prior to PDT (A, B) or at 72 h after PDT (C, D). *Insert*, the immunofluorescent staining pattern of individual cells was consistent with PD-1 expression on the plasma membrane. (E) PD-1 positive cells were counted in a time course analysis and reported per high-power field. Note the significant reduction in cell numbers caused by 5FU pretreatment prior to PDT, as seen at 1, 2, and 3 days post-PDT treatment (unpaired two-sided Student’s t-test), *P < 0.05; ***P < 0.005.

Tumors resistant to cancer therapies often thrive by upregulation of inhibitory factors that favor tumor growth in an immunosuppressive tumor microenvironment. Most tumors utilize neutralizing antitumor immunoregulatory signals to block the cytotoxicity of immune cells, resulting in immunotherapy failure. ICI has been developed over the past decade to counter these inhibitory factors, causing much excitement and optimism in the oncology world (2,28,75,76). Immune checkpoint molecules are inhibitory receptors present on the surface of both tumors and immune cells that inhibit immune recognition and T-cell-mediated killing, thereby allowing tumors to evade the immune system and undergo unrestrained tumor growth. Some of the most common immune checkpoint receptors are PD-1, PD-L1 and CTLA4 (2,22). Given the immune-activating effects of 5FU (alone and in combination with PDT) upon the recruitment of immune cells that control innate and adaptive immune responses, we investigated the effects of a 5FU combination on the expression of immune checkpoint markers. Murine AK lesions from the time course described in Figs. 1 and 2 were stained for PD-1 expression using immunofluorescence (Fig. 3). The number of PD-1 expressing cells rose from a very low level before PDT (Fig. 3A) to a ~15-fold induction at 72 h post-PDT (Fig. 3C and E), disappearing again by 1 week (Fig. 3E). PD-1+ cells are thought to be deleterious to the functioning of other antitumor immune components, *e.g*. by inhibiting cytotoxic CD8+ cells. Therefore, it is very interesting and potentially important that lesions treated with the 5FU-PDT combination compared to vehicle-only controls, showed a significant reduction in PD-1+ cells (Fig. 3C–E). The trend of reduced PD-1+ cells after 5FU was present even before PDT and became a large effect (relative reduction in PDT-stimulated PD-1+ cells of 43%) at 72 h post-PDT (Fig. 3E). One potential explanation for these results lies in the elevated inflammatory innate immune cell infiltrate observed in prior experiments. Increased inflammation may drive down PD-1 expression to ensure a strong cytotoxic T cell response. This apparent suppression of PD-1+ immune cells by pretreating lesions with 5FU prior to PDT provides a new avenue for exploration of ways to enhance the therapeutic benefit of PDT for pre-cancerous AK lesions.

## CONCLUSION

**Figure 4.**
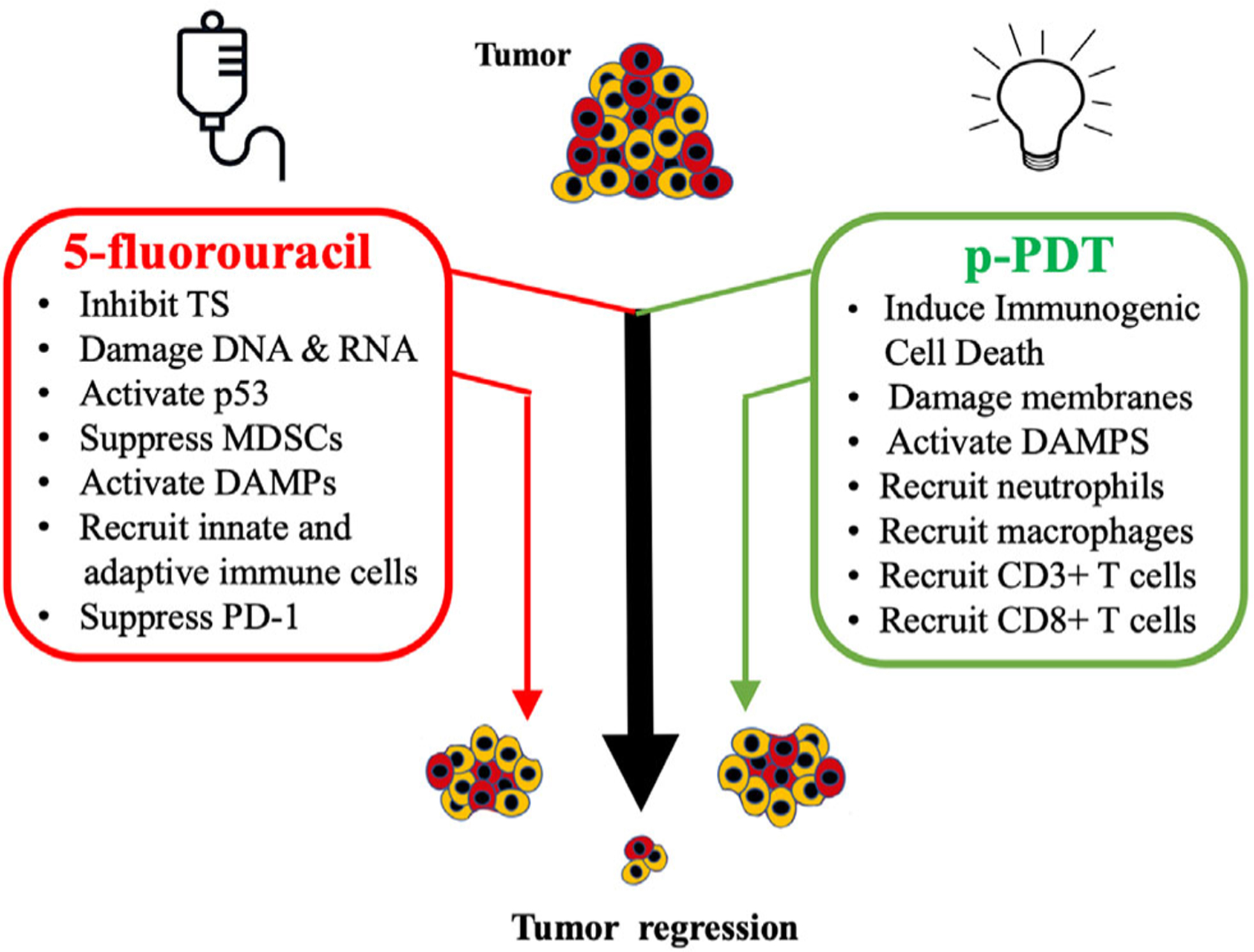
Summary: A combination of 5-fluorouracil with photodynamic therapy has additive effects upon antitumor immunity in a murine model of actinic keratosis. When applied to actinic keratoses (AK), 5FU exerts antimetabolite activities including inhibition of TS and damage to DNA and RNA through 5FU misincorporation. This results in activation of p53 and tumor cell death. In addition, 5FU treatment induces long-term antitumor immunity by suppressing myeloid-derived suppressor cells (MDSCs), activating DAMPs, recruiting innate (neutrophils and macrophages) and adaptive (CD3+ and CD8+) immune cell populations and suppressing programmed cell death-1 (PD-1), an immune checkpoint receptor on lymphocytes and other immune cells. Painless PDT (pPDT), when applied to 5FU-pretreated AK lesions, exerts additive effects on tumor eradication by inducing a second line of tumor-regressing response. The pPDT regimen, which induces ICD instead of the typical apoptotic cell death response observed after a conventional PDT regimen, results in activation of DAMPs and recruitment of cells comprising the innate (neutrophils and macrophages) and adaptive (CD3+ and CD8+) immune systems. In summary, a combination of 5FU and PDT, each individually known to induce long-term antitumor immune responses in addition to their more immediate antimetabolite and tumor cell death effects, work together and may synergize to provide better tumor eradication therapy.

The therapeutic advantage of 5FU as a neoadjuvant for ALA-PDT, as previously shown by us in preclinical and clinical studies, results from an augmentation of cell death induced by PDT *via* induction of differentiation-related pathways in cancer cells within the targeted epithelial tumors (8,48). Data presented in this study demonstrate an additional effect on the antitumor immune response observed when PDT is combined with 5FU pretreatment. In a time-course analysis, we have shown that a combination 5FU plus PDT, relative to vehicle plus PDT, leads to: (1) enhancement of an innate immune response characterized by higher numbers of neutrophils and macrophages within the lesion; (2) an improved adaptive response by recruitment of higher numbers of CD3+ and CD8+ T cells; and (3) suppression of the immune checkpoint marker PD-1. At the same time, ALA-PDT and 5FU treatment each result in an increased presence of macrophages and CD3+ T cells, and a decrease in PD1+ cells, when applied individually (Fig. 4). A detailed mechanistic investigation of the therapeutic potential for the 5FU-PDT combination therapy to achieve better management of human NMSCs *via* stimulation of an improved tumor-targeting immune response, is currently being explored.
